# Adipose-derived stem cells attenuate rheumatoid arthritis by restoring CX_3_CR1^+^ synovial lining macrophage barrier

**DOI:** 10.1186/s13287-025-04144-5

**Published:** 2025-03-05

**Authors:** Lei Wang, Ming Hao, Yongyue Xu, Zhaoyan Wang, Hanqi Xie, Bo Zhang, Xue Zhang, Jun Lin, Xiaodan Sun, Jianbin Wang, Qiong Wu

**Affiliations:** 1 MOE Key Laboratory of Bioinformatics, Center for Synthetic and Systems Biology, Tsinghua University, Beijing, 100084 China; 2 School of Life Sciences, Tsinghua University, Beijing, 100084 China; 3 Department of Orthopaedics, Suzhou Dushu Lake Hospital, The Fourth Affiliated of Soochow University, Medical Center of Soochow University, Suzhou, 215001 Jiangsu China; 4 School of Materials Science and Engineering, Tsinghua University, Beijing, 100084 China; 5 Key Laboratory of New Ceramics and Fine Processing, Tsinghua University, Beijing, 100084 China; 6 Key Laboratory of Advanced Materials of Ministry of Education of China, Tsinghua University, Beijing, 100084 China

**Keywords:** Mesenchymal stem cells, Rheumatoid arthritis, Macrophage, Mitochondria

## Abstract

**Background:**

Rheumatoid arthritis (RA) is a chronic autoimmune disease and the integrity of CX_3_CR1^+^ synovial macrophage barrier significantly impacts its progression. However, the mechanisms driving the dynamic changes of this macrophage barrier remain unclear. Traditional drug therapies for RA have substantial limitations. Mesenchymal stem cells (MSCs)-based cell therapy, especially adipose-derived stem cells (ADSCs), hold therapeutic promise. Nevertheless, the underlying therapeutic mechanism of ADSCs, especially their interactions with CX_3_CR1^+^ macrophages, require further investigation.

**Methods:**

To explore the interaction between ADSCs and CX_3_CR1^+^ synovial macrophages during barrier reconstruction, underlying the therapeutic mechanism of ADSCs and the mechanisms on the dynamic changes of the macrophage barrier, scRNA-seq analysis was conducted 4 days after ADSCs injection in serum transfer-induced arthritis model mice. The roles of mitochondria transfer and ADSCs transplantation were also explored. Bulk RNA-seq analysis was performed after the co-culture of ADSCs and CX_3_CR1^+^ synovial macrophages. To study the in vivo fate of ADSCs, bulk RNA-seq was performed on ADSCs retrieved at 0, 2, 4, and 7 days post-injection.

**Results:**

Intra-articular injection of ADSCs effectively attenuated the pathological progression of mice with serum transfer-induced arthritis. ADSCs gradually adhered to CX_3_CR1^+^ macrophages, facilitating the restore of the macrophage barrier, while the absence of this barrier greatly weakened the therapeutic effect of ADSCs. scRNA-seq analysis revealed an Atf3^high^ Ccl3^high^ subset of CX_3_CR1^+^ macrophages with impaired oxidative phosphorylation that increased during RA progression. ADSCs-mediated reduction of this subset appeared to be linked to mitochondrial transfer, and transplantation of isolated ADSCs-derived mitochondria also proved effective in treating RA. Both bulk RNA-seq and scRNA-seq analyses revealed multiple interaction mechanisms between ADSCs and CX_3_CR1^+^ macrophages, including Cd74/Mif axis and GAS6/MERTK axis, which contribute to barrier restoration and therapeutic effects. Furthermore, bulk RNA-seq analysis showed that ADSCs primarily contribute to tissue repair and immune regulation subsequently.

**Conclusions:**

Our results suggest that ADSCs ameliorated the energy metabolism signature of CX_3_CR1^+^ lining macrophages and may promote barrier restoration through mitochondria transfer. In addition, we elucidated the fate of ADSCs and the therapeutic potential of mitochondria in RA treatment.

**Supplementary Information:**

The online version contains supplementary material available at 10.1186/s13287-025-04144-5.

## Introduction

Rheumatoid arthritis (RA) is a common autoimmune disease. Despite its high incidence and morbidity, the exact etiology of RA remains unknown, among which macrophages play an important, dual role in the pathogenesis of RA [1, 2].

A subtype of tissue-resident macrophages with high expression of CX3CR1, TREM2, and tight junction proteins can form a protective barrier on the inner surface of the articular synovial membrane of mice [3]. This macrophage barrier is disrupted during RA, which leads to severe joint inflammation. Restoring this phagocytic barrier is critical for preventing RA relapses. Consistently, human synovial MERTK^+^ TREM2^+^ lining macrophage exhibit a similar structure, transitioning from a disrupted to a tight state during inflammation resolution and synovial repair [4]. Accordingly, the synovial lining macrophage barrier is a crucial driver and promising target for RA remission and potential cure [5]. Previously, we designed piezoelectric BaTiO3 ultrasound-driven nanorobots to repair the CX_3_CR1^+^ synovial macrophage barrier and alleviate RA symptoms [6]. However, the mechanisms underlying the dynamic changes of this barrier remain unclear.

At present, there is no cure for RA, non-steroidal anti-inflammatory drugs and disease-modifying antirheumatic drugs are commonly used to delay RA progression [7, 8]. However, 10–15% of RA patients still remain intolerant or resistant to these therapies [9]. In recent years, mesenchymal stem cells (MSCs)-based therapies, especially adipose-derived stem cells (ADSCs), have become a promising alternative for RA [10].

ADSCs have been shown to reduce synovial hyperplasia, pannus formation, invasion of joint structures and articular cartilage damage durin RA progression [10]. However, the dynamic changes in ADSC states post-implantation and their interactions with immune cells, particularly CX_3_CR1^+^ synovial lining macrophages, remain poorly understood.

MSCs can improve aerobic respiration and inhibit apoptosis of recipient cells through mitochondrial transfer [11–13]. Moreover, MSCs-based mitochondrial transplantation has been used to treat a variety of abnormal metabolism diseases [14]. In RA, abnormal oxidative phosphorylation and mitochondrial dysfunction disrupt immune homeostasis [15, 16], highlighting metabolic reprogramming as a key factor in RA pathogenesis [17]. Therefore, it is necessary to explore the therapeutic potential of ADSCs-derived mitochondria in RA treatment.

In this study, we explored the therapeutic effects and mechanisms of ADSCs in a mouse model of serum transfer-induced arthritis (STA), focusing on the dynamic regulation of the CX_3_CR1^+^ synovial macrophage barrier status. We evaluated the impact of ADSCs treatment on CX_3_CR1^+^ macrophages, the role of these macrophages in ADSCs-mediated therapy, and their interactions through co-culture experiments.

We further investigated the factors contributing to the disruption of CX_3_CR1^+^ macrophages, the mechanism by which ADSCs repair the barrier, their interactions with other synovial cells, including CX_3_CR1^+^ lining macrophages, and the in vivo fate of ADSCs at different stages through bulk RNA-seq and scRNA-seq analyses. From the perspectives of mitochondrial transfer and mitochondrial transplantation, we elucidated how ADSCs restore the CX_3_CR1^+^ macrophage barrier to exert their therapeutic effects.

This study offers promising insights and targets for the restoration of the CX_3_CR1^+^ macrophage barrier for RA treatment, highlighting energy metabolism as a key target and expanding the potential application of mitochondrial therapy in regenerative medicine. By elucidating the fate of ADSCs at different stages, it lays a robust theoretical and experimental foundation for further enhancing their therapeutic efficacy.

## Materials and methods

The work has been reported in line with the ARRIVE guidelines 2.0.

### Mouse strains and breeding

The KRN mice [18] used in this study were a kind gift from Prof. Benoist C. of Harvard University. NOD/LtJ mice were purchased from Gempharmatech Co., Ltd., Jiangsu, China. K/BxN mice were generated by crossing KRN and NOD/LtJ mice. CAG-EGFP mice, *Cx3cr1*^*cre*^*Rosa26*(*R26*)*-tdTomato* mice and *Cx3cr1*^*cre*^*R26-iDTR* mice were all based on the C57BL/6J background and were purchased from Shanghai Model Organisms Center, Inc., Shanghai, China. C57BL/6J mice were purchased from the Laboratory Animal Center of Tsinghua University, Beijing, China. All mouse strains were bred under specific pathogen free (SPF) conditions. All experimental procedures were in accordance with the requirements of animal welfare and ethics. All mice used in this study were aged between 6 and 18 weeks unless stated otherwise.

### Mouse anaesthesia and euthanasia

All the mice which need surgery were anesthetized by inhaling Isoflurane. K/BxN mice for serum extraction were anesthetized by intraperitoneal injection of Avertin (1.5 mL kg^− 1^). All the mice were euthanized using CO_2_ inhalation method in the animal center.

### Extraction, culture and identification of ADSCs

ADSCs were obtained from the inguinal fat of 10- to 14-day-old wild-type C57BL/6J mice and CAG-EGFP mice. Adipose tissues were digested incubated with 2 mg/mL collagenase II (Sigma-Aldrich, USA) and were separately resuspended in DMEM/F-12 complete medium. Unless explicitly stated otherwise, all experiments used EGFP-ADSCs for easy tracing.

The antibodies CD105-PE, CD29-APC, CD31-FITC, CD44-PerCP/Cyanine5.5, CD73-AF488 and SCAL-FITC (1:300–400, BioLgend, USA) were used to detect the expression of surface antigens by flow cytometry.

### RA model construction and scoring

The serum of K/BxN mice with significantly thickened paws was collected by cardiac blood collection method [18] and stored at − 80 ℃. The STA model was established by a single intraperitoneal injection of 200 µL K/BxN serum in healthy mice. Clinical development of arthritis was assessed using a clinical index ranging from 0 (weakest) to 16 (most severe), with a cumulative score for each paw ranging from 0 to 4, The scoring standard was referred to [3].

### Activation of cre and DT systems in transgenic mice

For activation of the cre system, the transgenic mice were administered 2 intraperitoneal injections of a total of 4 mg tamoxifen (MCE, USA) dissolved in peanut oil (Solarbio, China) within 48 h. For systemic administration of DT, mice were intraperitoneally injected with 100 µL of DT-PBS solution at a concentration of 5 mg/mL for 2 consecutive days, beginning 6 days before STA induction.

### In vivo injection of ADSCs

15 µL of PBS or cell suspension containing 6 × 10^5^ ADSCs (passages 4 to 6 ) was injected into each of the two knee cavities of each mouse. For TNT inhibition in ADSCs, ADSCs were treated with 10 µM nocodazole (Biyuntian, China) for 6 h, after which the cells were washed 3 times with PBS.

### Co-culture experiments

Normal co-culture system: 3.6 × 10^4^ tdTomato labeled Ly6G^−^ CD45^+^ CD11b^+^ F4/80^+^ CX_3_CR1^+^ synovial macrophages sorted through flow cytometry and 3.6 × 10^4^ EGFP-labeled ADSCs were added to a 48-well culture plate. Observations and imaging recordings were performed under a fluorescence microscope (Thermo Fisher, USA) after 6/24/48/72/96 h of co-culture.

Co-culture in Transwell chambers: 1 × 10^5^ ADSCs were added to the Transwell chamber membrane (polycarbonate, 0.4 μm pore size) in a 12-well plate, and 1 × 10^5^ CX_3_CR1^+^ macrophages were added to the well under the chamber. The cells were observed and imaged under a fluorescence microscope after 24/48/72/96 h of co-culture.

Co-culture system of mitochondria-labeled cells: 1 × 10^4^ ADSCs labeled with MitoTracker-Red or MitoTracker-Green was co-cultured with 1 × 10^4^ CX_3_CR1^−^ synovial macrophages; 1 × 10^4^ EGFP-Cox8-ADSCs and 1 × 10^4^ synovial tdTomato-CX_3_CR1^+^ macrophages were added to the confocal dish. Observation was conducted after 24 h under the 60× objective of a spinning-disk confocal microscope (Dragonfly, Andor, UK).

### Extraction and culture of synovial cells from knee joints

Operations were conducted as described before [3]. The digested cells were resuspended in RPMI 1640 complete medium for culture. Fresh complete medium was added on the third day, and half of the medium was replaced every 2 days. Numerous synovial cells could be observed after 7–10 days of cell adhesion.

### HE and SO/FG staining of paraffin-embedded tissues sections

The knee and ankle joints of mice were clipped and fixed in 4% PFA-PBS solution (Leagene, China) for 12 h at 4 °C. After decalcification for 10 days, the tissues were embedded, dehydrated, sectioned, stained, and sealed according to the conventional hematoxylin-eosin (HE) and safranin O–fast green (SOFG) staining procedures. The sections were scanned and imaged using the Pannoramic SCAN slide scanning system (3DHISTECH, Hungary).

### Tissue cryosections of knee joints

The mouse joints were cut and immediately fixed in 4% PFA-PBS solution at 4 °C for 12 h. The cryosections were generated as described in [19]. Sections with a thickness of 8 μm were prepared and stored at − 80 °C.

### Immunofluorescence staining and observation

Sections blocking was conducted as described before [3]. F4/80-APC or CD68-FITC fluorophore conjugated antibodies or anti-rabbit Atf3, Immt or Cox5a unconjugated primary antibodies (Bioss, China) were added at 1:300 or 1:400 dilutions in staining solution (BioLegend, USA) and separately incubated overnight at 4 °C. Donkey anti-rabbit IgG AF488 secondary antibody (Beyotime, USA) was added correspondingly for Atf3, Immt and Cox5a staining for 4 h at room temperature. The samples were washed with DPBS for 3 times and sealed by adding anti-fluorescence attenuation sealant containing DAPI (Solarbio, China) and kept at 4 °C in the dark. After 4 h, the slides were observed under a spinning-disk confocal microscope.

### Sorting of synovial macrophages

Cells blocking was conducted as described before [3]. The blocked cells were stained with fluorophore-conjugated antibodies, including CD11b-PeCy7, CD45-BV421, and F4/80-FITC (exclude for sorting scRNA-seq cells) and Ly6G-AF647 (1:300–400, BioLegend, USA) for 25 min at 4 °C in the dark. After washing with PBS, the cells were resuspended in PBS containing 2% FBS. Ly6G^−^ CD45^+^ CD11b^+^ F4/80^+^ cells were sorted into RPMI 1640 medium containing 1% FBS for in vitro experiments. Ly6G^−^ CD45^+^ CD11b^+^ cell subsets and EGFP-labeled ADSCs were sorted for scRNA-seq.

For the retrieval of injected ADSCs in vivo, the whole knee joint were removed and cut into small pieces and placed into DMEM/F-12 digestion medium containing 10% FBS, 2 mg/mL collagenase IV from *Clostridium histolyticum* and 0.03 mg/mL DNase I. After 60–75 min of digestion, the samples were resuspended as descried above. EGFP-labeled cells were sorted for Smart-Seq2 library construction and sequencing.

### Enzyme-linked immunosorbent assay

Serum was collected from mice as described above for KB×N serum. IL-6, IL-10 and IL-1β levels were measured according to the Mouse IL-6 Uncoated ELISA kit (Invitrogen, USA), Mouse IL-10 Uncoated ELISA kit (Invitrogen, USA), and mouse IL-1β Uncoated ELISA kit (Invitrogen, USA), separately.

### Real-time quantitative PCR

To assess the gene expression levels, the total RNA of mouse knee joint synovium tissue was extracted using TRIZOL reagent (Invitrogen, USA) and reverse-transcribed into cDNA using the SuperScript II First-Strand Synthesis System Kit (GenStar, China). The RT-PCR reactions were conducted using 2 × RealStar Green Fast Mixture (GenStar, China). *GAPDH* served as the internal reference gene and data were analyzed using the 2^−△△CT^ method. The specific primers were as follows: *TNF-α* (sense, 5′-CAT CTT CTC AAA ATT CGA GTG ACA A-3′; anti-sense, 5′-TGG GAG TAG ACA AGG TAC AAC CC-3′), *IL-1β* (sense, 5′-AGG TCG CTC AGG GTC ACA AG-3′; anti-sense, 5′-GTG CTG CCT AAT GTC CCC TTG AAT C-3′), and *GAPDH* (sense, 5′-GTA GTT GAG GTC AAT GAA GGG-3′; anti-sense, 5′-TCG TCT CAT AGA CAA GAT GGT-3′).

### Labeling, extraction, observation and injection of mitochondria

MitoTracker-Red or Green fluorescence stain (Beyotime, China) was used at a dilution of 1: 2000 to label the mitochondria. Mitochondria extraction was conducted using mitochondrial extraction kit (Solarbio, China) and the isolated mitochondria were labeled with MitoTracker-Green for observation. During the extraction process, the samples must be kept at 0–4 °C to maintain the viability of the mitochondria. The isolated mitochondria were resuspended in mitochondria storing buffer and 15 µL of the storing buffer or mitochondria extracted from 6 × 10^5^ ADSCs were injected into each knee joint cavity of mice.

### Lentivirus production and transfection

The N-terminal mitochondrial mapping sequence of COX8 (MSVLTPLLLRGLT) was obtained from NCBI and attached to the N terminus of the EGFP expression box in the pCDH EGFP lentiviral plasmid.

The EGFP-Cox8 plasmid was introduced into HEK293T cells together with the psPAX2 and pMD2.G plasmids using Lipofectamine 3000 (Invitrogen, USA) to produce lentiviral particles. The filtered concentrated lentivirus and 8 mg/L polybrene (Sigma-Aldrich, USA) were added to the ADSCs culture medium, and the successfully transfected cells were subsequently selected using 2 mg/L puromycin (Beyotime, China).

### Bulk RNA sequencing

Each parallel group contained 100 sorted cells. Bulk RNA sequencing was conducted according to the Smart-Seq2 protocol [20] as described previously.

### Single-cell RNA sequencing

GEM generation, cDNA synthesis and library construction were performed using a 10X Chromium Single Cell 3′ Solution v2 single cell RNA sequencing kit (10X Genomics, USA) according to the manufacturer’s manual. Library sequencing was performed on an Illumina HiSeq 6000 platform to a depth of 1 × 10^8^ reads per sample to achieve approximately 70% sequencing saturation.

### RNA-seq data processing

Bulk NGS data were mapped to the mouse reference genome (mm10, Ensembl annotation release 91) using Hisat2 v2.1.0 [21], and qualified using Qualimap v2.2.1 [22]. Data with mapping rates below 60% or exonic rates below 60% were discarded. Transcript quantification was performed using Salmon v0.8.2 [23]. PCA dimension was calculated using the prcomp function in R v4.3.1 (R Foundation for Statistical Computing, Vienna, Austria), and the outliers were filtered before downstream analysis. The differential expression test was performed using the DEseq2 package [24] in R.

Single-cell NGS data were mapped and quantified using cellranger v7.0.1 (10X Genomics) in intron-included mode. Reads were then aligned to the mouse reference genome (mm10, Ensembl annotation release 91) including the additional annotation for EGFP and tdTomato. The count matrix was normalized, integrated and clustered using the Seurat V4 package [25] in R. Then differentially expressed genes were identified using the FindMarker function in Seurat. The tSNE and heatmap were visualized using functions integrated in Seurat.

Pathway enrichment analysis was conducted using the clusterProfiler package [26] in R with differential expressed genes calculated from bulk and single-cell sequencing data. Enrichment results were visualized using the ggplot2 package [27] in R.

Cell interactions were estimated using the CellChat package [28], and visualized using the circlesize package [14] in R.

### Statistical analysis

All the experiments were performed in at least three independent replicates. Quantitative data were presented as means ± SD using Microsoft Excel 2023 (Microsoft Corp., USA) and visualized with GraphPad Prism 10.0 (GraphPad Software Inc., USA). The statistical significance of differences was assessed using two-way ANOVA followed by Tukey’s multiple comparisons tests. Differences with P-values of less than 0.05 were considered significant. Asterisks were used to indicate significance as follows: * *P* < 0.05, ** *P* < 0.01, *** *p* < 0.001, **** *p* < 0.0001.

The confocal laser scanning microscopy imaging results were processed using Imaris Viewer 10.1 (Oxford Instruments plc., UK). The scanning results of paraffin sections were viewed and processed using Pannoramic Viewer 1.15.4 (3DHISTECH Ltd., HU). The flow cytometry results were processed using FlowJo 10.0 (Becton, Dickinson & Compan, USA). SnapGene 5.3.1 (GSL Biotech LLC, USA) was used to process the plasmid sequences. Cell length, area and fluorescence intensity data were calculated by ImageJ 1.54j (Wayne Rasband, USA).

Visualized results of bulk RNA-seq and scRNA-seq analyses were mainly generated by Seurat and CellChat, or the circlesize and ggplot2 packages in R. In Seurat-based gene expression analysis, the Wilcoxon rank-sum test was applied to identify differentially expressed genes. The results of gene set enrichment analysis based on clusterProfiler were statistically verified using the Benjamini-Hochberg test.

## Results

### Articular injection of ADSCs attenuated the progression of rheumatoid arthritis in mice

**Fig. 1:**
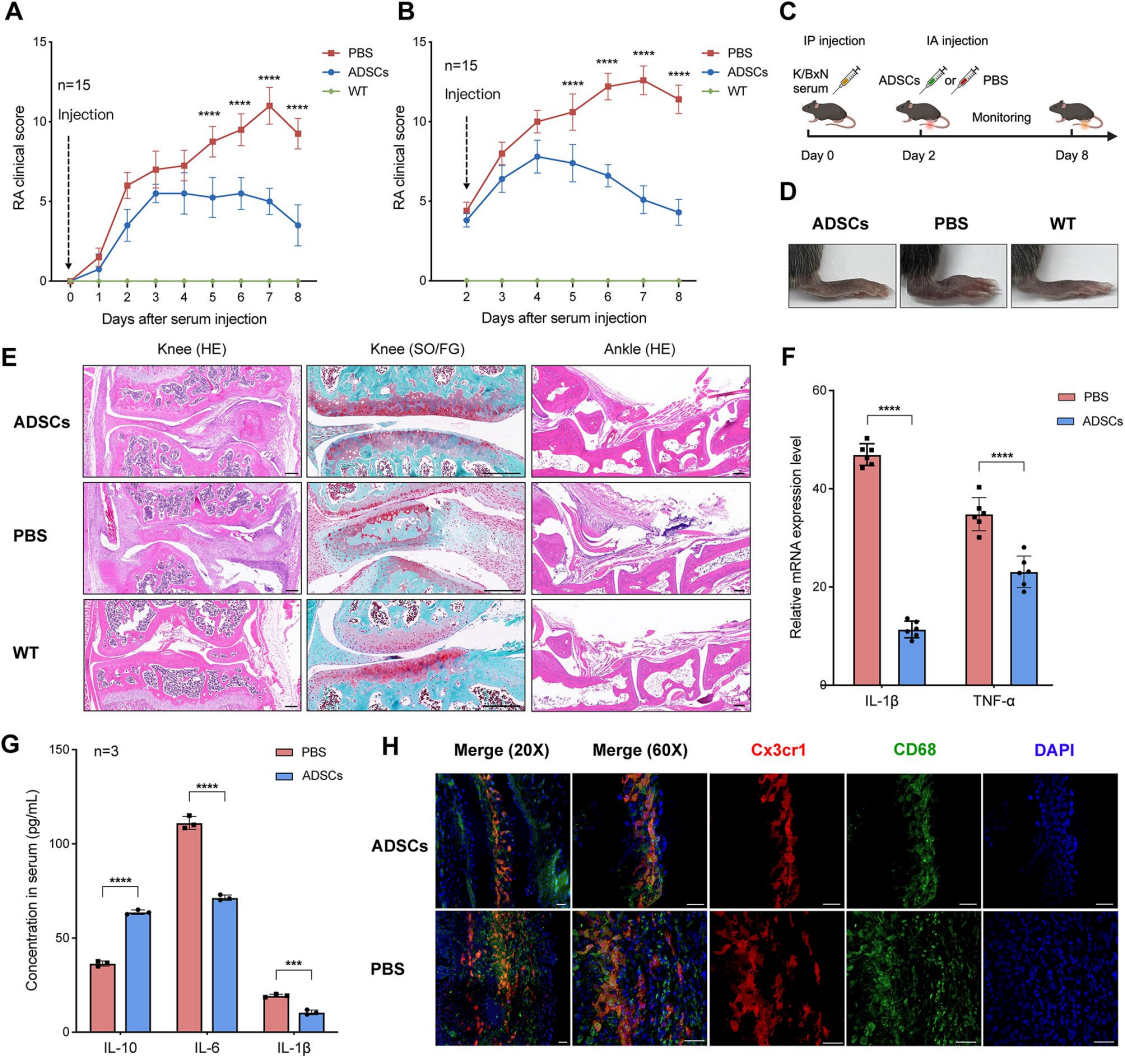
Intra-articular injection of ADSCs mitigated RA and restored the CX_3_CR1^+^ macrophage barrier in mice STA model. **(A)** RA clinical score when injecting ADSCs on day 0. **(B)** RA clinical score when injecting ADSCs on day 2. **(C)** Flow diagram of the in vivo injection experiment. **(D)** Swelling degree of mouse hind paws on day 8. **(E)** HE and SO/FG stanning results of histological sections of mouse knee and ankle joints on day 8. Scale bar, 200 μm. **(F)** The mRNA levels of pro-inflammatory factors in knee joint synovium on day 8. **(G)** The levels of pro-inflammatory and anti-inflammatory factors in peripheral blood on day 8. **(H)** Cryosection immunofluorescence stanning of CX_3_CR1^+^ lining macrophages in the knee joints of ADSCs and PBS injected mice using confocal laser scanning microscopy (CLSM) on day 8. Scale bar, 30 μm

Stem cells were defined based on the minimal criteria established by the International Society for Cellular Therapy (Fig. S1). To visualize CX_3_CR1^+^ lining macrophages, we used *Cx3cr1*^*cre*^*Rosa26*(*R26*)*-tdTomato* mice in all experiments unless otherwise specified. ADSCs were directly injected into the articular cavities of both knees on day 0 immediately after K/BxN serum injection. The negative control group received an equivalent PBS injection. Clinical scoring showed that ADSCs significantly attenuated RA progression, with marked improvement in pathological severity observed one week post-injection (Fig. 1A).

To better simulate clinical scenarios, ADSCs were injected on day 2, when the CX_3_CR1^+^ macrophage barrier had already been disrupted according to Culemann et al. [3] and our own findings. Even under these conditions, ADSCs exerted therapeutic effects (Fig. 1B-D). Histological staining of knee and ankle joints on day 8 showed reduced joint inflammation and cartilage damage in the ADSCs-treated group compared to the PBS group (Fig. 1E). ADSCs also downregulated *IL-1β* and *TNF-α* expression in the knee joint synovium (Fig. 1F). ELISA results further revealed increased systemic IL-10 levels and decreased IL-6 and IL-1β levels in the ADSCs-treated group (Fig. 1G).

These results indicate that intra-articular ADSCs injections in the STA model significantly lower RA clinical scores, suppress pro-inflammatory cytokines, and mitigate neutrophil infiltration and cartilage erosion. Consequently, day 2 injections were selected for all subsequent experiments unless otherwise specified.

### Interaction between ADSCs and CX_3_CR1^+^ lining macrophages in vivo

To explore the impact of ADSCs treatment on the CX_3_CR1^+^ macrophage barrier, we performed confocal immunofluorescence microscopy on knee joints from ADSCs- and PBS-treated mice on day 8. Synovial tdTomato^+^ macrophages co-stained with CD68 were identified as CX_3_CR1^+^ macrophages (Fig. 1H). After ADSCs treatment, the macrophage barrier showed a dense, healthy-like state, while the PBS-treated group displayed a disrupted barrier.

**Fig. 2:**
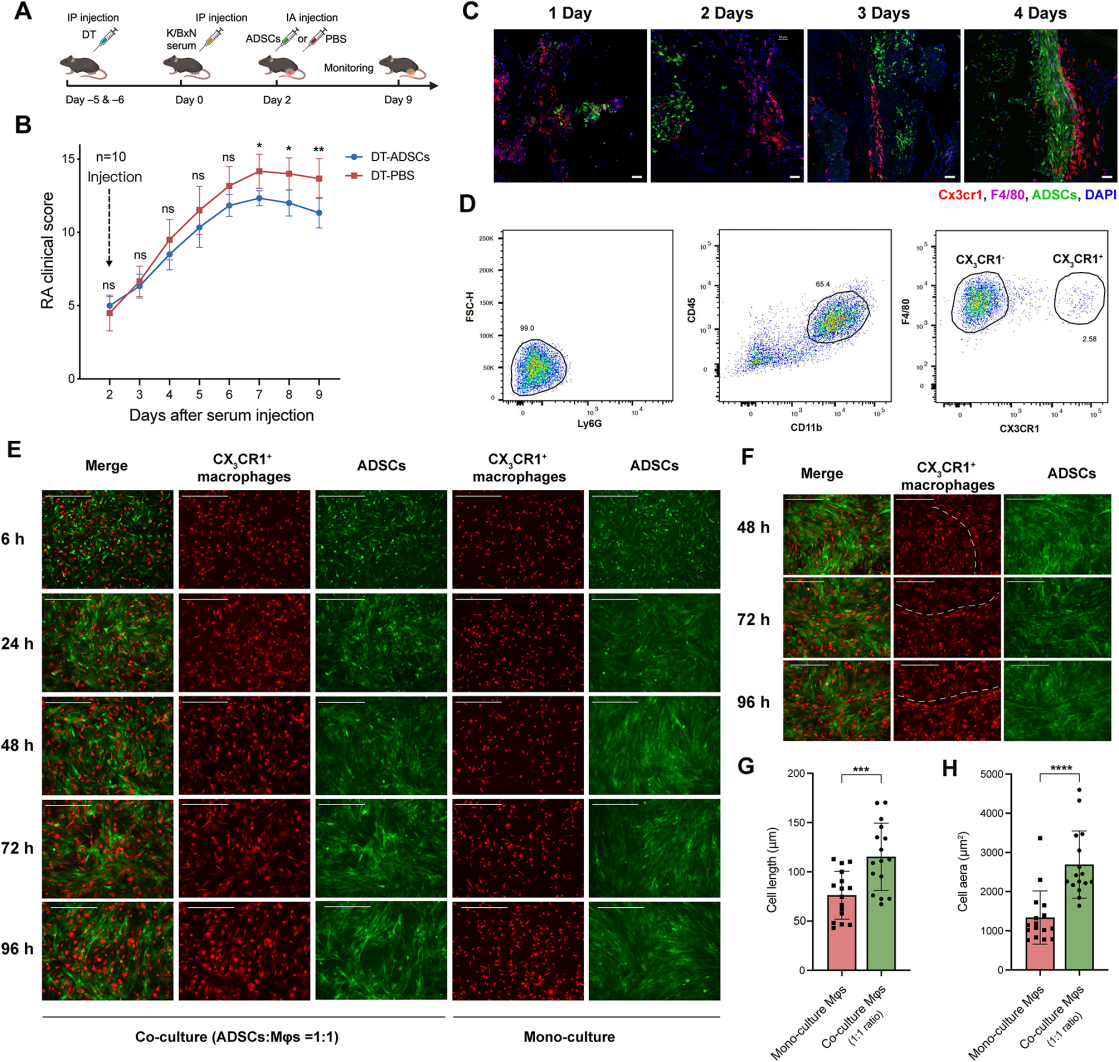
ADSCs and CX_3_CR1^+^ lining macrophages co-localize both in vivo and in vitro. **(A)** Flow diagram of the strategy used to deplete CX_3_CR1^+^ lining macrophages and in vivo injection experiment. **(B)** RA clinical score after the depletion of CX_3_CR1^+^ lining macrophages. **(C)** Immunofluorescence stanning observation of the localization of ADSCs and CX_3_CR1^+^ lining macrophages in knee joints using CLSM on days 1–4 after ADSCs injection (on days 3–6). Scale bar, 50 μm. **(D)** Flow cytometry sorting of CX_3_CR1^+^ lining macrophages from animal tissues. **(E)** Co-culture of CX_3_CR1^+^ lining macrophages and ADSCs at a 1:1 ratio. Scale bar, 400 μm. **(F)** The spontaneously formed linear structure of CX_3_CR1^+^ lining macrophages in the co-culture system at a 1:1 ratio. Scale bar, 400 μm. **(G-H)**. Cell length and cell area of CX_3_CR1^+^ lining macrophages in different culture system

To investigate the role of CX_3_CR1^+^ lining macrophages in the ADSCs treatment process, we crossed *Cx3cr1*^*cre*^ mice with mice containing a Cre-inducible diphtheria toxin receptor (*Cx3cr1*^*cre*^*R26-iDTR* mice), enabling diphtheria toxin (DT)-mediated depletion of synovial CX_3_CR1^+^ macrophages as described in [3]. On day 2, mice received a single articular injection of PBS or ADSCs, and RA clinical scores were assessed. Depletion of CX_3_CR1^+^ macrophages exacerbated arthritis progression and diminished the therapeutic effects of ADSCs (Fig. 2A-B). These results indicate that ADSCs promote the reconstruction of the CX_3_CR1^+^ macrophage barrier and these macrophages are pivotal for ADSCs-mediated RA treatment.

Since ADSCs were injected directly into the joint cavity, and CX_3_CR1^+^ macrophages form the innermost synovial layer, we examined the spatial localization of ADSCs post-injection by cryosection microscopy. Between 1 and 4 days post-injection, many ADSCs gradually adhered to the synovial lining layer, establishing direct contact with CX_3_CR1^+^ macrophages. During this period, the CX_3_CR1^+^ macrophage barrier gradually transitioned to a denser state (Fig. 2C). This suggests a close interaction between ADSCs and CX_3_CR1^+^ macrophages in the therapeutic process.

### Cell-cell communication between ADSCs and CX_3_CR1^+^ synovial lining macrophages in vitro

To explore the interaction between ADSCs and CX_3_CR1^+^ macrophages, we isolated CX_3_CR1^+^ synovial lining macrophages from healthy mice via flow cytometry (Fig. 2D) and conducted in vitro co-culture and transwell culture experiments. In the co-culture system with a 1:1 ratio, CX_3_CR1^+^ macrophages showed enhanced adhesion and cell elongation compared to those cultured alone (Fig. 2E), suggesting an enhanced anti-inflammatory phenotype [29, 30]. After 72 h of co-culture, the macrophages spontaneously formed band-like structures (Fig. 2F), and the two cell types formed cavity-like co-localization structure (Fig. 2E). These co-localization structures became more pronounced with a 2:1 ratio of ADSCs to macrophages (Fig. S2). However, these phenomena were absent in the transwell culture system, which physically separates the two cell types (Fig. S3), indicating that direct contact is crucial for these effects.

**Fig. 3:**
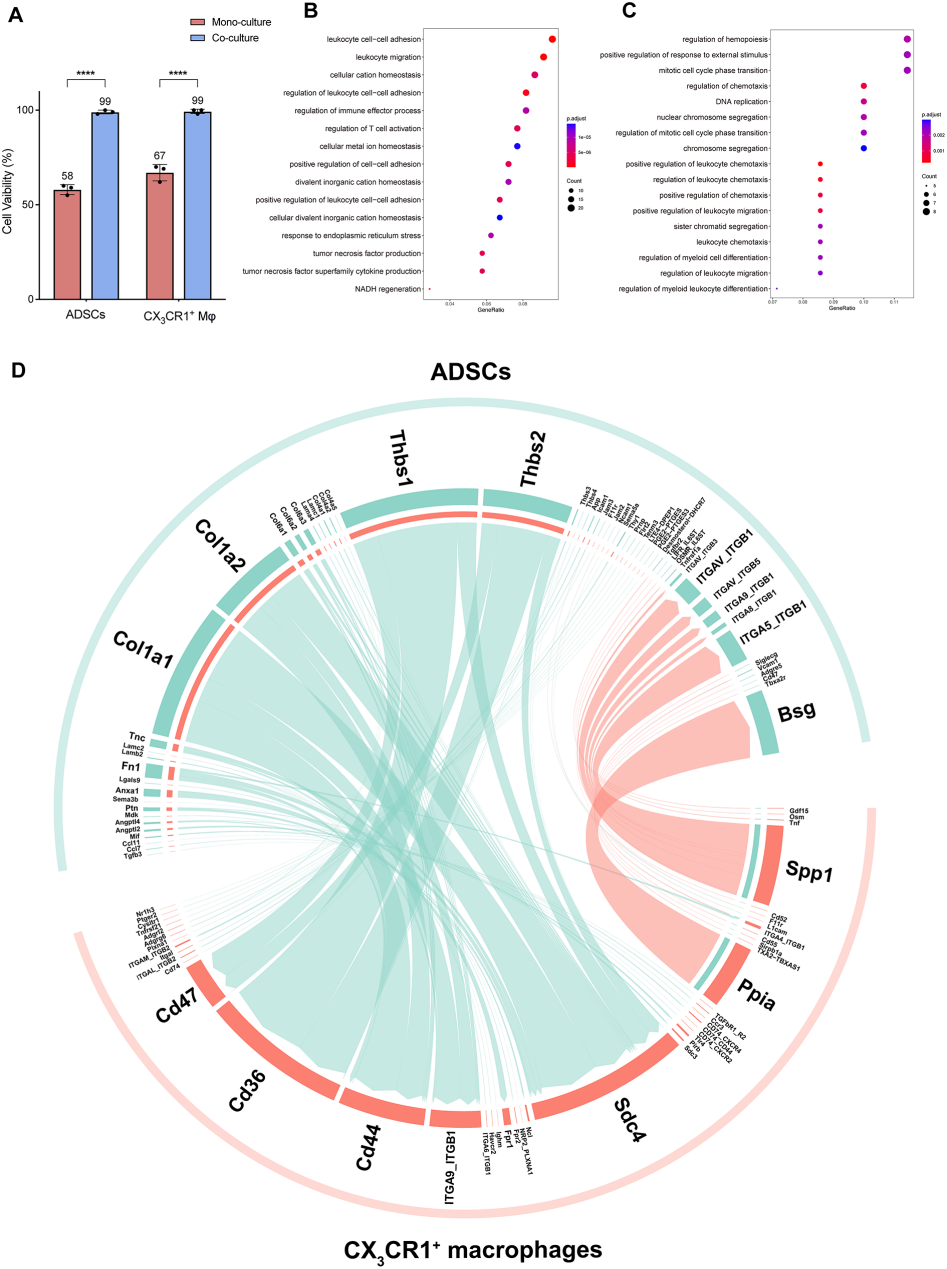
Bulk transcriptome changes of ADSCs and CX_3_CR1^+^ lining macrophages after co-culture. **(A)** Cell viability difference between separately cultured and direct co-cultured cells after 96 h at 1:1 ratio. **(B)** GO analysis of upregulated genes in co-cultured ADSCs vs. separately cultured ADSCs (log2FC > 1, Padj < 0.05). **(C)** GO analysis of upregulated genes of co-cultured CX_3_CR1^+^ lining macrophages vs. individually cultured CX_3_CR1^+^ lining macrophages (log2FC > 1, Padj < 0.05). **(D)** CellChat chord diagram of cell-cell communication between ADSCs and CX_3_CR1^+^ lining macrophages based on the bulk RNA-seq data (Padj < 0.05)

After 96 h of co-culture at a 1:1 ratio, ADSCs and macrophages were sorted by flow cytometry. Cell viability of both cell types significantly increased in co-culture compared to mono-culture according to trypan blue staining (Fig. 3A). Bulk-transcriptome sequencing of sorted cells revealed notable changes in both ADSCs and CX_3_CR1^+^ macrophages. In co-cultured ADSCs, Gene Ontology (GO) enrichment analysis identified terms related to the mitotic cell cycle, DNA replication, and chromosome segregation as significantly upregulated, indicating enhanced proliferation (Fig. 3B). Additionally, terms related to leukocyte chemotaxis regulation, migration, and differentiation were enriched. Cx3cl1, the ligand for CX3CR1, was also upregulated in co-cultured ADSCs. The CX3CL1-CX3CR1 axis plays an important role in regulating leukocyte chemotaxis and adhesion [31]. These results indicate that ADSCs could enhance cell proliferation as well as regulate the chemotaxis, migration and differentiation of macrophages in the co-culture environment.

In co-cultured CX_3_CR1^+^ macrophages, GO terms related to leukocyte cell-cell adhesion, migration, and regulation of immune effector processes were significantly enriched and upregulated (Fig. 3C). Among them, the expression of *Rac2* and *Ccl2* increased, suggesting polarization of macrophages toward an anti-inflammatory M2 phenotype [32]. Moreover, tight junction-related genes, including *Tjp1* and *F11r*, were significantly upregulated, indicating enhanced barrier function in CX_3_CR1^+^ macrophages.

The CellChat analysis revealed a sophisticated cell-cell communication network between ADSCs and CX_3_CR1^+^ synovial macrophages(Fig. 3D). Paracrine signaling from ADSCs, such as Col1a1 and Col1a2 interacting with Cd44, Sdc4, and ITGA9_ITGB1 on macrophages, promotes cell adhesion and migration. Additionally, direct cell-cell contact via Thbs1 and Thbs2 interacting with Cd47 and Cd36 from macrophages influences apoptosis, TGF-β activation, and immune regulation. While the Cd200-Cd200R axis enhances macrophage anti-inflammatory activity.

Ppia produced by CX_3_CR1^+^ macrophages could interact with Bsg on ADSCs to promote cell migration, proliferation and differentiation. Spp1 from CX_3_CR1^+^ macrophages could interact with integrin family proteins on ADSCs to regulate cell adhesion and migration.Notably, upregulation of Mif-Cd74 axis between ADSCs and CX_3_CR1^+^ macrophages restricts ADSCs migration, enhancing their homing ability [33].

These findings demonstrate that ADSCs and CX3CR1^+^ synovial macrophages interact through multiple mechanisms to promote cell adhesion, differentiation, migration, chemotaxis, and proliferation. This interplay accounts for key observations, including the gradual adhesion of ADSCs to CX_3_CR1^+^ synovial macrophages in vivo and the enhanced adhesion and spontaneous formation of tightly connected structures by co-cultured CX_3_CR1^+^ macrophages in vitro.

### The causes of CX_3_CR1^+^ synovial lining macrophage barrier disruption

**Fig. 4:**
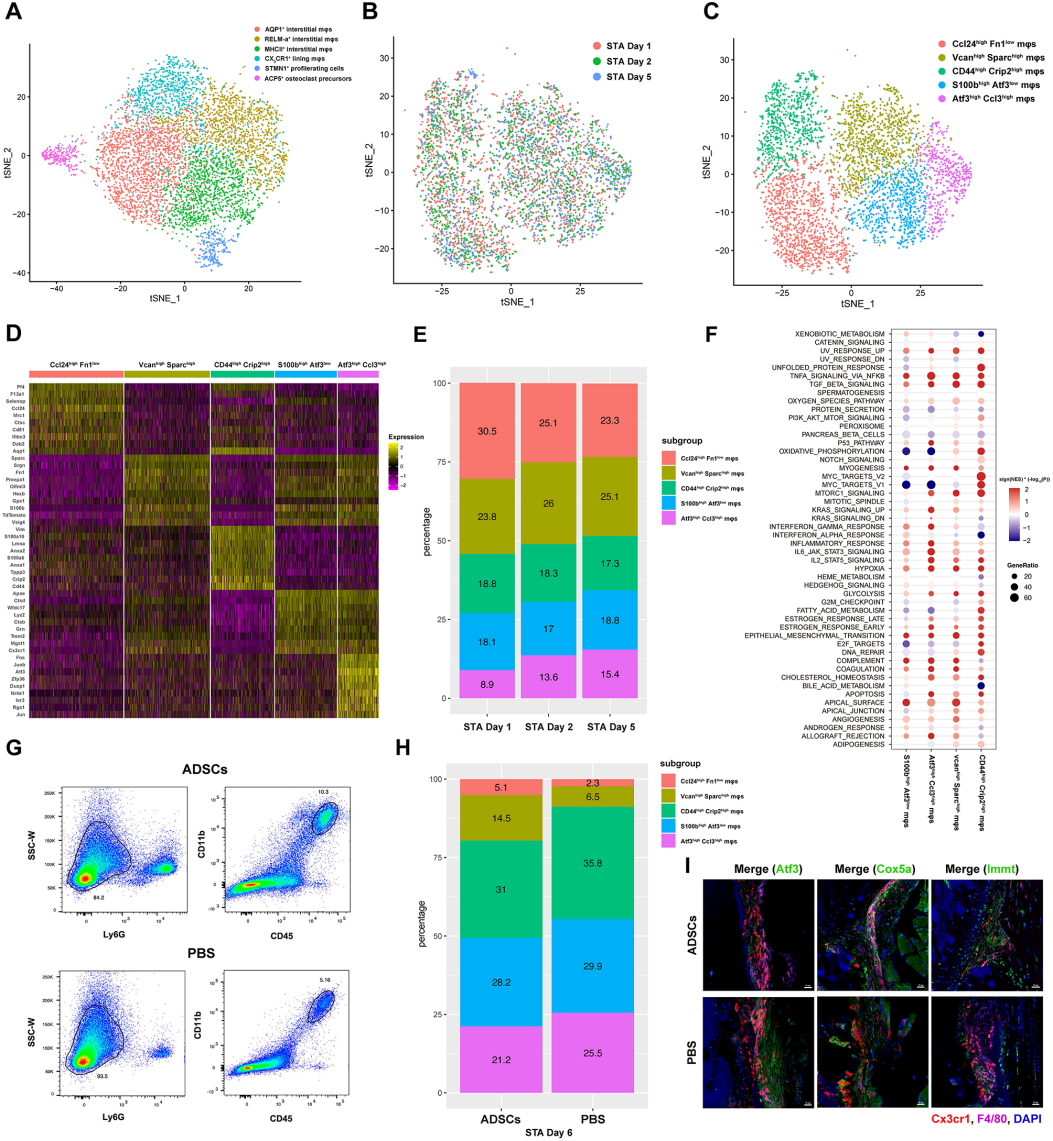
ScRNA-seq analysis on the heterogeneity change of CX_3_CR1^+^ synovial lining macrophages in the barrier disruption and restoration process. **(A)** Annotated tSNE map of the subpopulations of synovial CD45^+^ CD11b^+^ LY6G^−^ cells. **(B-C)** tSNE map of CX_3_CR1^+^ lining macrophage subpopulations on STA days 1, 2, and 5. **(D)** Heatmap of differentially express genes in five subpopulations of CX_3_CR1^+^ lining macrophages (Padj < 0.05, top 10 most significantly differentially expressed). **(E)** The ratio of five cell subpopulations on STA days 1, 2, and 5. **(F)** GSEA analysis results showing hallmark changes of four cell subpopulations compared to Ccl24^high^ Fn1^low^ macrophages (Padj < 0.05). **(G)** Flow cytometry strategy for sorting synovial CD45^+^ CD11b^+^ LY6G^−^ cells. **(H)** The relative ratios of five cell subpopulations between the ADSCs and PBS injection groups. **(I)** Atf3/Cox5/Immt immunofluorescence stanning of knee joints of ADSCs and PBS injected mice on day 8

During the gradual destruction of the CX_3_CR1^+^macrophage barrier, we noticed significant spatial rearrangement of some cells, while others retained their original positions, suggesting potential heterogeneity in the cell population. To investigate this, we reanalyzed scRNA-seq data of CD45^+^ CD11b^+^ Ly6G^−^ synovial monocytes from Culemann et al. [3] (Fig. 4A). CX_3_CR1^+^ lining macrophages from day1/2/5 of the STA model were combined and re-clustered into five distinct subsets, including Ccl24^high^ Fn1^low^, Vcan^high^ Sparc^high^, Cd44^high^ Crip2^high^, S100b^high^ Atf3^low^, and Atf3^high^ Ccl3^high^ (Fig. 4B-D, S4A). During the progression of RA, the proportion of Ccl24^high^ Fn1^low^ macrophages showed a downward trend, whereas Atf3^high^ Ccl3^high^ macrophages increased, while the other three subsets showed no siginificant changes (Fig. 4E).

Consistent with findings by Culemann et al. and our immunofluorescence microscopy observations, the CX_3_CR1^+^ macrophage barrier became less cohesive and was eventually destroyed as RA progressed. This was associated with a depletion of macrophages in a relatively normal physiological state, accompanied by an expansion of cells in a pathological state. Accordingly, we hypothesized that Ccl24^high^ Fn1^low^ macrophages represent a physiological cell subset, while Atf3^high^ Ccl3^high^ macrophages are indicative of a pathological state.

To explore the biological functions of the five cell subsets, we performed gene set enrichment analysis (GSEA), using Ccl24^high^ Fn1^low^ macrophages as the reference to calculate the log fold change (LFC) (Fig. 4F). The results showed that the oxidative phosphorylation pathway and MYC target V1 related pathway were significantly downregulated in pathological Atf3^high^ Ccl3^high^ macrophages compared with the other subsets. In contrast, inflammation-related pathways, such as IFN response and IL6-JAK-STAT3 signaling, were significantly upregulated. MYC, a well characterized transcription factor and oncogene, is known to promote cell growth and proliferation [34]. The hypoxic environment of RA-affected joints disrupts cellular metabolism, and the function of inflammatory macrophages are believed to be driven by metabolic activity that is biased toward glycolysis and pentose phosphorylation pathways due to inefficient mitochondrial ATP production. These findings revealed that the Atf3^high^ Ccl3^high^ macrophages represent a proinflammatory subpopulation with impaired oxidative phosphorylation, supporting our hypothesis.

Oxidative phosphorylation is kown to affects the integrity of tight junctions, with its impairment in epithelial cells leading to tight junction dysfunction and subsequent damage to intestinal and blood-brain barriers. Therefore, the disrupted oxidative phosphorylation of CX_3_CR1^+^ macrophages may also cause the destruction of tight junctions, leading to the breakdown of the synovial barrier and the loss of its protective function.

### Effects of ADSCs treatment on the heterogeneity of CX_3_CR1^+^ synovial lining macrophages

To further investigate the therapeutic effect of ADSCs on the CX_3_CR1^+^ lining macrophage barrier and their in vivo interactions, we sorted synovial CD45^+^ CD11b^+^ Ly6G^−^ monocytes from the ADSCs-treated and PBS-treated groups four days post-injection to conduct scRNA-seq (Fig. 4G). The analysis revealed and increased proportion of Ccl24^high^ Fn1^low^ macrophages, indicative of a physiological state, and a reduced proportion of Atf3^high^ Ccl3^high^ macrophages, indicative of the pathological state, in the ADSCs-treated group compared to the PBS group (Fig. 4H). These results suggest that the restoration of a tight macrophage barrier following ADSCs injection may be attributed to improved oxidative phosphorylation metabolism in macrophages, promoting the expansion of normal subsets while reducing pathological subsets.

These findings were verified by immunofluorescence staining of joint sections, which showed decreased Atf3 expression in CX_3_CR1^+^ lining macrophages post-ADSCs treatment. The expression of Cox5a and Immt, key regulators of oxidative phosphorylation, was upregulated, further supporting the metabolic restoration hypothesis (Fig. 4I) [35, 36].

CellChat analysis revealed a robust communication network between ADSCs and synovial CD45^+^ CD11b^+^ Ly6G^−^ cell subsets, with the strongest interactions occurring between ADSCs and CX_3_CR1^+^ lining macrophages (Fig. S4B). Predicted interactions indicated that extracellular matrix proteins such as Col1a1, Col1a2, Col6a1, Col6a2, and Col6a3 secreted by ADSCs interacted with Cd44 and ITGA9_ITGB1 on these cells to mediate cell-cell migration and adhesion. Additionally, pleiotrophin (Ptn), a growth and angiogenesis factor secreted by ADSCs, was predicted to exert paracrine effects on CX_3_CR1^+^ lining macrophages, CX_3_CR1^−^ RELMα^+^ macrophages, and STMN1^+^ proliferative cells. Based on its known capacity to stimulate the proliferation of human synovial cells and monocytic cell line THP-1, Ptn may promote the proliferation of these subpopulations. In addition, all cell subsets interacted with ADSCs through Ppia, which binds Bsg on ADSCs to mediate cell migration, proliferation and differentiation.

The communication between ADSCs and CX_3_CR1^+^ lining macrophages was predominantly initiated by ligands emitted by ADSCs (Fig. S4B), aligning with observations from in vitro co-culture experiments. Specifically, Col1a1, Col1a2, Col6a1, Col6a2, and Col6a3 interacted with Cd44, Sdc4, and ITGA9_ITGB1 on CX_3_CR1^+^ lining macrophages, enhancing cell-cell adhesion and migration. Other significant interactions included Fn1-Cd44/ITGAV_ITGB1, Postn-ITGAV_ITGB5, and Ptn-Ncl, which regulate cell adhesion, migration, proliferation, and differentiation.

ADSCs were also found to signal through Gas6, interacting with Mertk on CX3CR1^+^ lining macrophages. The MERTK/GAS6 axis is known to suppress the production of proinflammatory cytokines, promoting anti-inflammatory effects [4]. MERTK^+^ macrophages were previously reported to be driven by GAS6 to induce a repair phenotype in lining fibroblasts [37].

Consistent with the results of in vitro co-culture experiments, ADSCs could promote the polarization of macrophages towards an anti-inflammatory phenotype. This polarization likely involves a metabolic shift, as inflammatory macrophages typically rely on glycolysis and the pentose phosphate pathway for energy production, rather than mitochondrial oxidative phosphorylation [38]. ADSCs appear to restore oxidative phosphorylation in CX_3_CR1^+^ lining macrophages, potentially improving their barrier function.

These results suggest that ADSCs contribute to the repair of the synovial barrier by restoring the oxidative phosphorylation of CX_3_CR1^+^ lining macrophages. In addition, ADSCs regulate macrophage migration, adhesion, chemotaxis, and differentiation through multiple signaling pathways that contribute to mitigating RA pathology.

### The roles of ADSCs mitochondria in RA treatment

**Fig. 5:**
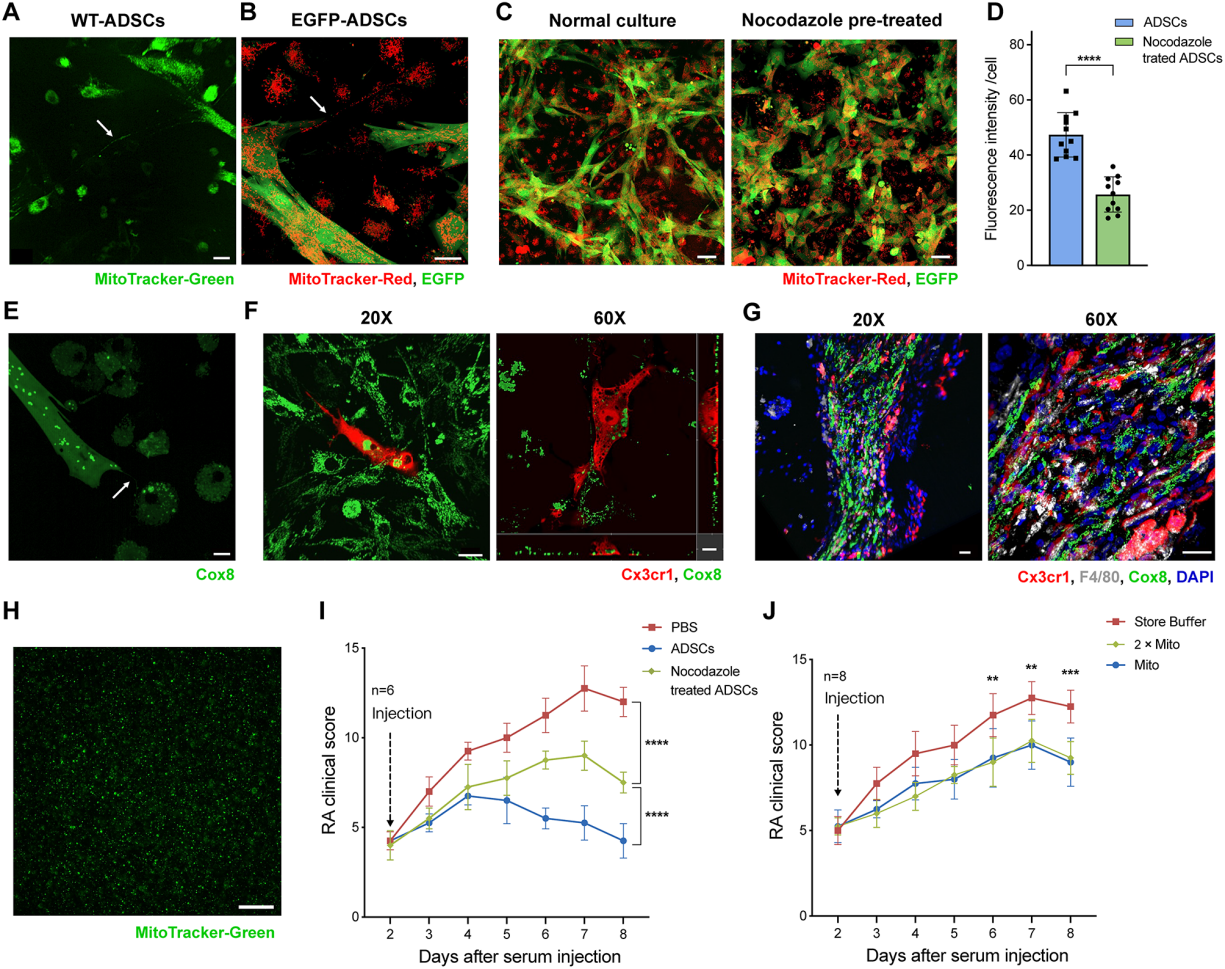
Mitochondrial transfer from ADSCs during the RA treatment process. **A-E** used CX_3_CR1^−^ synovial macrophages and **F** used CX_3_CR1^+^ synovial macrophages. **(A)** Transfer of mitochondria from WT-ADSCs (no fluorescence) stained with MitoTracker-Green to CX_3_CR1^−^ synovial macrophages in vitro. Scale bar, 20 μm. **(B)**. Transfer of mitochondria from EGFP-ADSCs (EGFP fluorescence) stained with MitoTracker-Red to CX_3_CR1^−^ synovial macrophages in vitro. Scale bar, 20 μm. **(C)** Inhibition of mitochondrial transfer from ADSCs by pretreatment with 10 µM nocodazole for 6 h. Scale bar, 40 μm. **(D)** Red fluorescence intensity of single CX_3_CR1^−^ lining macrophage in different groups. **(E)** Mitochondrial transfer from EGFP-Cox8-ADSCs to CX_3_CR1^−^ macrophages. Scale bar, 15 μm. **(F)** Mitochondrial transfer from EGFP-Cox8-ADSCs to CX_3_CR1^+^ lining macrophages. Scale bar, 30 & 10 μm, from left to right. **(G)** CLSM observation of mitochondrial transfer from EGFP-Cox8-ADSCs to synovial cells in vivo on STA day 4. Scale bar, 20 μm. **(H)** Isolated mitochondria of ADSCs stained with MitoTracker-Green. Scale bar, 100 μm. **(I)** RA clinical score changes when the TNTs of ADSCs were inhibited using nocodazole. **(J)** The role of the mitochondrial transplantation from ADSCs in RA treatment

Oxidative phosphorylation occurs on the inner mitochondrial membrane, which can be affected by mitochondrial morphology, number and composition. To examine the role of mitochondrial transfer from ADSCs in RA treatment, we investigated the transfer of mitochondria from ADSCs to macrophages. WT-ADSCs (non-fluorescent) and EGFP-ADSCs were labeled with MitoTracker-Green or Red, respectively, to distinctly track mitochondria without interference from cellular fluorescence. After 24 h of co-culture with CX_3_CR1^−^ synovial macrophages, labeled mitochondria were detected within macrophages, and the formation of tunneling nanotubes (TNTs) were observed (Fig. 5A-B). When ADSCs were pretreated with 10 µM of the TNT inhibitor nocodazole for 6 h, the number of labeled mitochondria in macrophages decreased, indicating that mitochondrial transfer is mediated through TNTs (Fig. 5C-D).

To avoid false-positive results caused by dye leakage, we constructed a lentiviral vector encoding EGFP fused to the mitochondria locating sequence of Cox8 at N-terminal (EGFP-Cox8) to specifically label the mitochondria of ADSCs. The mitochondria of EGFP-Cox8-ADSCs exhibited fluorescence, and mitochondrial transfer was comfirmed in co-culture experiments (Fig. 5E). When EGFP-Cox8-ADSCs were co-cultured with CX_3_CR1^+^ synovial lining macrophages, a certain number of mitochondria were observed in macrophages (Fig. 5F). Further more, 2 days after intra-articular injection of EGFP-Cox8-ADSCs, fluorescence-labeled mitochondria were detected inside synovial macrophages in cryosections of knee joints (Fig. 5G). To test the functional relevance of this process, ADSCs were treated with 10 µM nocodazole for 6 h to inhibit mitochondrial transfer. This treatment significantly reduced the therapeutic efficacy of ADSCs, demonstrating that mitochondrial transfer is critical for the therapeutic effect (Fig. 5I).

To investigate the therapeutic potential of mitochondrial transplantation, we directly injected fresh mitochondria extracted from 6 × 10^5^ ADSCs into the joint cavities of mice (Fig. 5H). Although mitochondrial transplantation showed a modest therapeutic effect, doubling the dose of mitochondria did not significantly improve the outcome, indicating a limited role in RA treatment (Fig. 5J).

Combined with scRNA-seq analysis, these findings indicate that ADSCs enhance the oxidative phosphorylation of CX_3_CR1^+^ synovial lining macrophages partly through mitochondrial transfer, contributing to their therapeutic effect in RA.

### In vivo fate of ADSCs at different stages of RA treatment

**Fig. 6:**
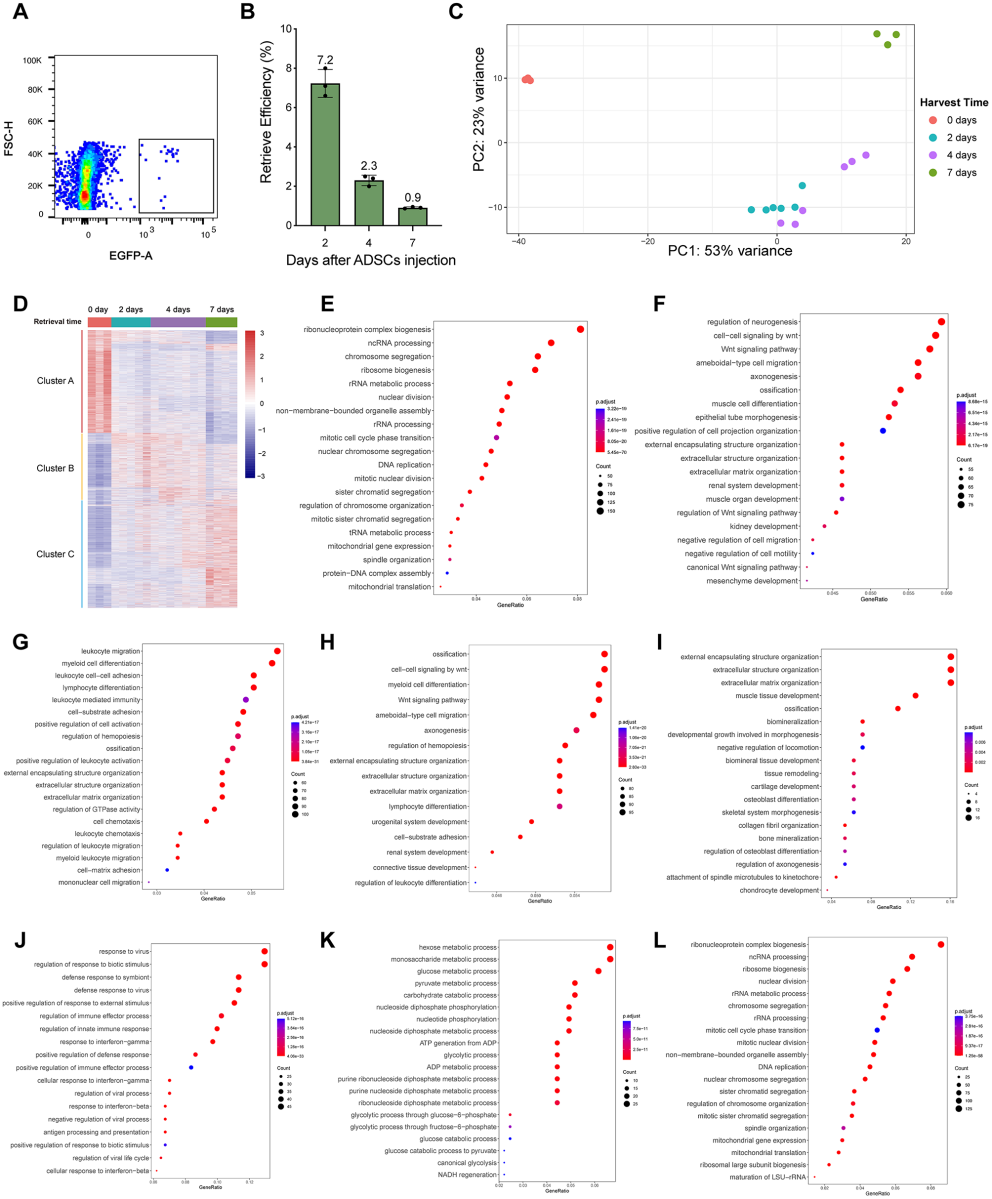
In vivo fate of ADSCs during RA treatment. **(A)** ADSCs were sorted by flow cytometry. **(B)** ADSCs retrieval efficacy on days 2, 4, and 7. **(C)** PCA analysis of ADSCs retrieved at different timepoints. **(D)** Heatmap of differentially expressed genes among ADSCs retrieved at different days. **(E)** GO analysis of gene cluster A. **(F)** GO analysis of gene cluster B. **(G)** GO analysis of gene cluster C. **(H-L)** Enriched GO terms of upregulated genes in **(H)** 2-day-ADSCs vs. 0-day-ADSCs, **(I)** 4-day-ADSCs vs. 2-day-ADSCs, **(J)** 7-day-ADSCs vs. 4-day-ADSCs, and downregulated genes in **(K)** 7-day-ADSCs vs. 2-day-ADSCs, **(L)** 7-day-ADSCs vs. 0-day-ADSCs. Differentially Expressed genes included in **E-L** were screened using the criteria log2FC > 1, Padj < 0.05

To investigate the in vivo fate of ADSCs, cells were retrieved by flow cytometry at 2, 4, and 7 days after intra-articular injection, and their bulk transcriptomic profiles were analyzed at each timepoints, along with pre-injection ADSCs. The retrieval rate of ADSCs decreased raplidly over time, from 7.2% by 2 days to only 0.9% by 7 days after injection (Fig. 6A-B). PCA analysis showed that only 2- and 4-day group were of high similarity (Fig. 6C).

Differentially expressed genes (DEGs) analysis identified three clusters associated with the time since injection (Fig. 6D). Cluster A genes, characterized by a monotonous decrease in expression, were primarily involved in RNA and ribosome synthesis, DNA replication, and mitochondrial translation, processes closely linked to cell proliferation (Fig. 6E). Cluster B genes, which exhibited an initial increase in expression followed by a slight decline, were enriched for pathways related to Wnt signaling, ossification, cell migration, and extracellular matrix organization. The Wnt signaling pathway is known to regulate MSCs proliferation, self-renewal, and differentiation (Fig. 6F). Cluster C genes, with monotonously increasing expression, were associated with leukocyte migration, intercellular adhesion, differentiation and immune modulation, cell-substrate adhesion, osteogenesis, and extracellular matrix organization (Fig. 6G).

These findings suggest that ADSCs exert their therapeutic effects in RA joints by enhancing stem cell differentiation, upregulating factors involved in immune regulation, and enhancing cell adhesion to interact more effectively with CX_3_CR1^+^ macrophages.

Moreover, ADSCs played different dominant functions at different treatment stages. At 2 days post-injection, compared to pre-injection ADSCs, processes such as ossification, Wnt signaling, extracellular structures and matrix organization, and leukocyte differentiation were significantly upregulated (Fig. 6H).

By 4 days post-injection, extracellular structure and matrix organization, ossification, negative regulation of cell migration, and tissue remodeling were dominant compared to the 2-day timepoint. At this stage, biological processes such as cartilage and chondrocyte development, osteoblast differentiation, and collagen fiber assembly were initiated. These changes corresponded to enhanced extracellular matrix formation and reduced migration of ADSCs, consistent with the observed adhesion to CX_3_CR1^+^ lining macrophages (Fig. 6I).

By 7 days post-injection, ADSCs shifted their primary role towards immunomodulation, as evidenced by the upregulation of genes related to antiviral responses, immune regulation, IFN-γ response, and antigen processing and presentation, compared to the 4-day timepoint (Fig. 6J). In RA, IFN-γ, secreted by various cell types, is known to improve the anti-inflammatory ability of MSCs [39], suggesting that ADSCs played a central role in regulating joint inflammation at this stage.

When comparing the 7-day group with the pre-injection and 2-day groups, genes related to cell proliferation and energy metabolism were downregulated, indicating that cellular activity of ADSCs was inhibited in the nutrient-deprived knee joint cavity. These findings demonstrate that ADSCs adapt their functions over time, transitioning from promoting extracellular matrix organization and tissue remodeling to predominantly immunomodulatory roles as the treatment progresses.

## Conclusions

In this study, we demonstrated that intra-articular injection of ADSCs attenuates RA progression and promotes the reconstruction of the CX_3_CR1^+^ synovial macrophage barrier in STA model mice. Experimental abrogation of this barrier greatly reduced the therapeutic effect of ADSCs. Using bulk RNA-seq in vitro and scRNA-seq in vivo, we analyzed the interaction mechanisms between ADSCs and CX_3_CR1^+^ macrophages, revealing enhanced cell-cell adhesion, migration, chemotaxis, proliferation, and differentiation—processes critical for macrophage barrier repair.

scRNA-seq analysis revealed a distinct subset of CX_3_CR1^+^ macrophages with impaired oxidative phosphorylation and heightened inflammatory responses that increased during RA progression. This subset’s metabolic dysfunction may be linked to tight junction degradation, as supported by prior research [40]. ADSCs repaired the macrophage barrier by transferring mitochondria to CX_3_CR1^+^ synovial macrophages and depleting this metabolically impaired subset, thereby exerting their therapeutic effect. Inhibition of mitochondrial transfer diminished the efficacy of ADSCs, while direct intra-articular transplantation of ADSCs-derived mitochondria also showed therapeutic potential in RA. In addition, bulk transcriptomic analysis of ADSCs at different time points post-injection revealed their leading roles in early differentiation, tissue repair, and immune regulation.

## Discussion

### STA as a model for MSCs-based treatment of RA

In 2022, a research team administered 0.5 × 10^6^ ADSCs per mouse by intraperitoneal injection on day 1 of the STA model, but failed to achieve the desired therapeutic effect [41]. In our study, we injected a total of 1.2 × 10^6^ ADSCs into two knee joint cavities of mice on day 0 or day 2 of the STA model, resulting in a significant therapeutic effect on RA.

We speculate that this difference is primarily due to variations in stem cell dose and delivery method, as these factors have also led to inconsistent outcomes in other RA models [42, 43]. Following K/BxN serum injection, G6PI IgG antibodies rapidly accumulate in the joints, initiating downstream immune responses which lead to rapid disease onset [44]. Given that only a small proportion of cells injected intraperitoneally or intravenously reach the joint cavity ultimately, direct intra-articular injection appears to be a more effective delivery approach. The rapid onset of the STA model and its focus on the arthritic effector phase make it a convenient system for exploring the therapeutic mechanisms of MSCs. Our findings highlight the importance of optimizing cell delivery methods and doses to enhance the therapeutic efficacy of MSCs in different RA models.

### Relationship between cellular metabolism and the disruption of the CX_3_CR1^+^ macrophage barrier

This study demonstrated that ADSCs injection enhances the oxidative phosphorylation capacity of CX_3_CR1^+^ synovial lining macrophages. This finding was initially inferred from scRNA-seq analysis and preliminarily validated through cryosection immunofluorescence staining. However, further investigation, including metabolic functional assays such as Seahorse analysis, is required to ascertain the effects of ADSCs and their mitochondria on oxidative phosphorylation in these macrophages.

Additionally, the relationship between oxidative phosphorylation and tight junction status in CX_3_CR1^+^ macrophages warrants further exploration. Nevertheless, evidence from other inflammatory conditions provides potential insights. In inflammatory bowel disease, the dysfunction of oxidative phosphorylation and fatty acid β-oxidation in mitochondria has been linked to abnormal tight junction of intestinal epithelial cells, leading to barrier dysfunction [40]. Similarly, treating Madin-Darby canine kidney (MDCK) cells with an oxidative phosphoric acid uncoupler increased intercellular permeability, which was reversed upon its removal, allowing tight junction proteins to redistribute [45].

In our previous work, we developed piezoelectric tetragonal BaTiO_3_ ultrasound-driven nanorobots for controllable electrical stimulation to repair the CX_3_CR1^+^ macrophage barrier [6]. Piezoelectric stimulation could reprogram macrophages, while BaTiO_3_ scaffolds inhibit inflammatory MAPK/JNK signaling cascade and activate oxidative phosphorylation and ATP synthesis in macrophages [46]. Based on this study, BaTiO_3_ scaffold may exert its regenerative effect by improving oxidative phosphorylation of CX_3_CR1^+^ macrophages.

In addition, the fatty acid metabolism of Atf3^high^ Ccl3^high^ macrophages was significantly less active than that of other macrophages subsets (Fig. 4F). Given the dual role of lipid metabolism in macrophage polarization [47], investigating how different metabolic phenotypes influence cellular functions in vivo could uncover valuable therapeutic targets for RA.

### Application prospects of MSCs mitochondria

In this study, we used nocodazole to inhibit TNT formation and mitochondrial transfer. However, as nocodazole disrupts microtubule dynamics, it may also partially impair the biological function of ADSCs, potentially influencing their therapeutic effects. Additionally, inhibiting mitochondrial transfer affects the process between ADSCs and various cell types in vivo. A key limitation is our inability to selectively inhibit mitochondrial transfer to CX_3_CR1^+^ synovial lining macrophages. A deeper understanding of the mechanisms underlying mitochondrial transfer initiation and delivery could help address this challenge.

At present, the mechanisms driving active mitochondrial transfer from donor to recipient cells remain unclear. It is speculated that impaired energy metabolism of recipient cells or injury-related factors on the cell surface, such as phosphatidylserine, may induce TNT formation and promote mitochondria transfer to damaged cells. Studies have shown that Miro1 and connexin 43 is crucial for TNT formation and mitochondrial transfer [48, 49]. In this study, the mitochondrial transfer process from ADSCs to CX_3_CR1^+^ macrophages may rely on similar mechanism, but whether specific induction and regulation mechanisms are employed by different cell types requires further investigation.

Based on existing findings, mitochondrial transfer from MSCs can be regulated in various ways, and their mitochondria can be modified to enhance functionality. These approaches offer promising opportunities to expand the applications of mitochondrial transfer in regenerative medicine.

### Enhancing the therapeutic efficacy of ADSCs

Our findings reveal that, beyond mitochondria transfer and inflammatory phenotype polarization, ADSCs and CX_3_CR1^+^ synovial lining macrophages interact through enhanced cell adhesion and migration. This interaction may lead to the spontaneous formation of band-like structures by CX_3_CR1^+^ lining macrophages in co-culture systems. In vivo, the attachment of ADSCs to the synovial lining may facilitate the migration and subsequent adhesion of CX_3_CR1^+^ macrophages to the synovium, contributing to the repair of the macrophage barrier.

Despite the fact that intra-articular ADSCs injection has a therapeutic efficacy in alleviating RA pathology, some limitations still remain. The cells may secrete pro-inflammatory factors (e.g. CCL10 and CCL6), potentially hindering RA remission and repair. RNA-seq analysis indicated that prolonged residence in the synovial cavity appears to compromise the energy supply of ADSCs. Future strategies could focus on genetically engineering ADSCs to suppress negative factor expression while enhancing their migration, adhesion, and energy supply to improve therapeutic outcomes.

In conclusion, this study provide a promising target for the reconstruction of the CX_3_CR1^+^ macrophage barrier and the treatment of inflammatory diseases such as RA from the perspective of metabolism. It also expands the prospects of mitochondrial therapy in regenerative medicine. Exploring the mechanism of the treatment effect of ADSCs in RA also provides an experimental and theoretical basis for future engineering of ADSCs.

## Electronic supplementary material

Below is the link to the electronic supplementary material.

Supplementary Material 1

## Data Availability

The bulk and single-cell RNA-seq data that support the findings of this study has been uploaded onto GEO DataSets (accession number GSE273883) and other information are available from the corresponding author upon reasonable request.

## Declarations

### Generative AI in scientific writing

The authors did not use generative AI in completing this manuscript.
