# Supplementary material for: Adipose-derived stem cells attenuate rheumatoid arthritis by restoring CX3CR1+ synovial lining macrophage barrier
Source: Stem Cell Res Ther. 2025 Mar 5;16:111. doi: 10.1186/s13287-025-04144-5 (PMC11881422; doi:10.1186/s13287-025-04144-5)
Supplement: Supplementary file 1 — Supplementary Material 1 [file 13287_2025_4144_MOESM1_ESM.docx]

Supplementary Materials for

**Adipose-derived Stem Cells Attenuate Rheumatoid Arthritis by**

**Restoring CX_3_CR1^+^ Synovial Lining Macrophage Barrier**

Lei Wang *et al.*

*Corresponding author. Email: wuqiong@tsinghua.edu.cn

**This PDF file includes:**

Figs. S1 to S4


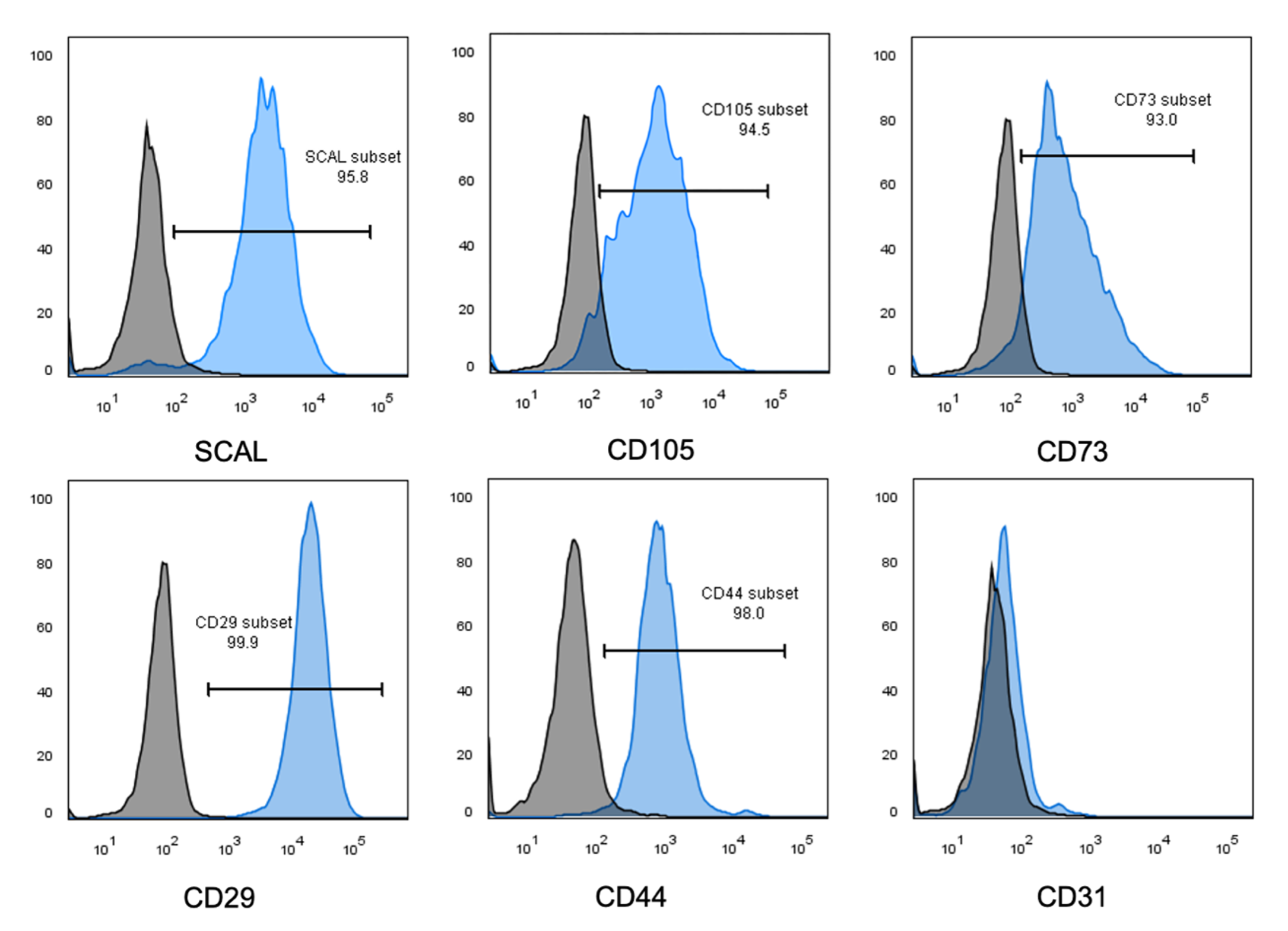


Fig. S1.

Identification of the surface markers of ADSCs by flow cytometry.


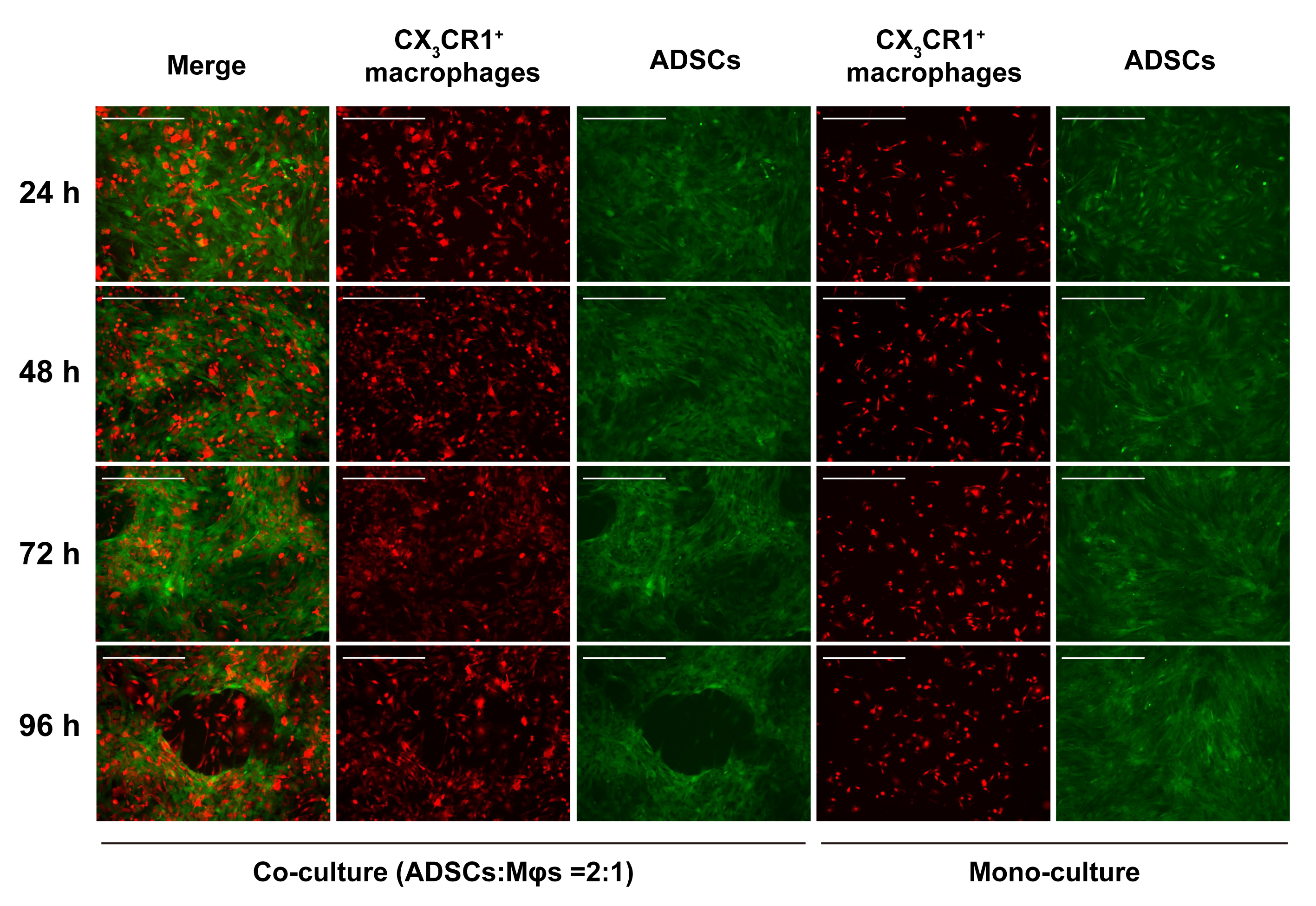


Fig. S2.

Co-culture of ADSCs and CX_3_CR1^+^ lining macrophages at a 2:1 ratio. Scale bar, 400 μm.


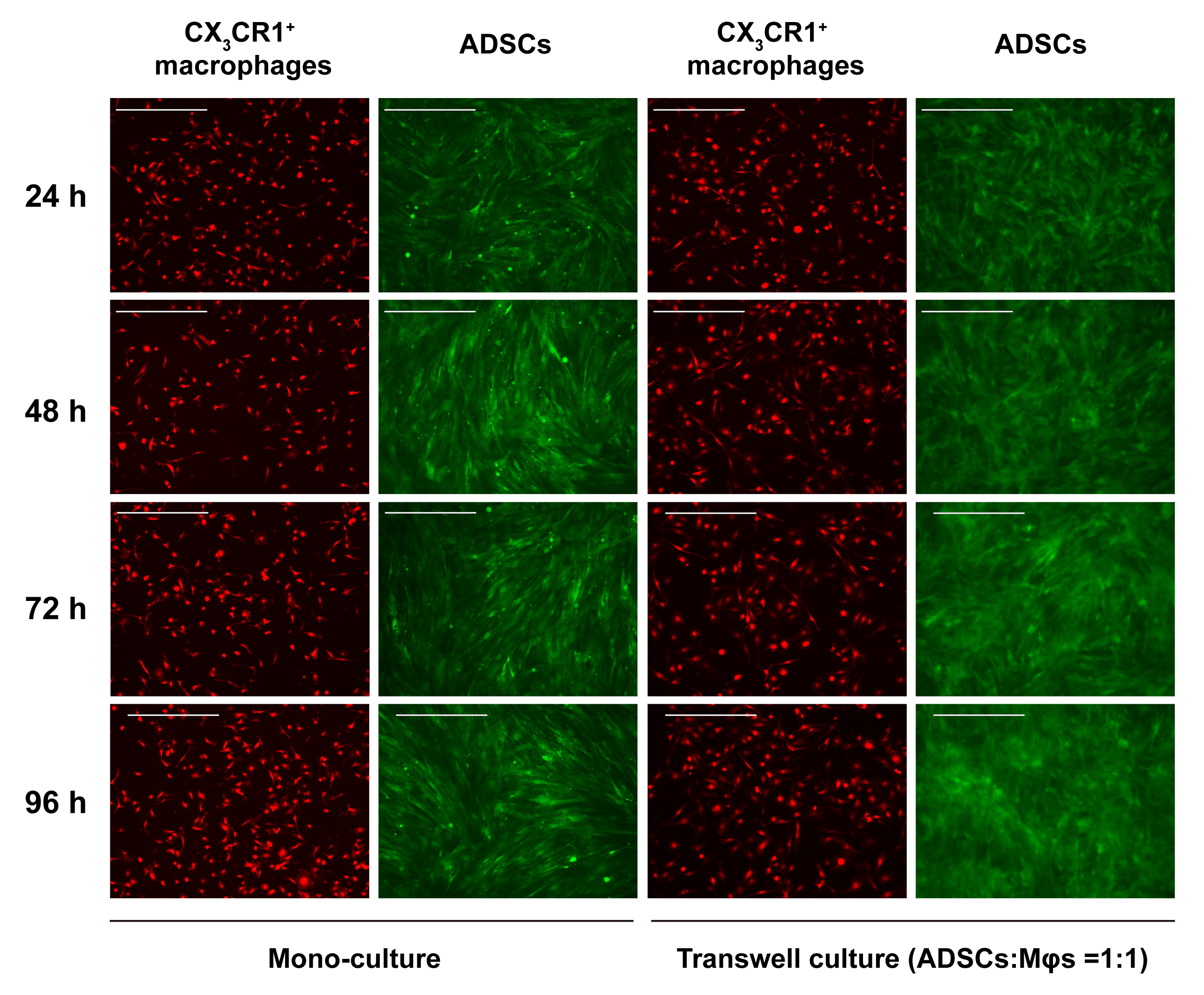


Fig. S3.

Transwell culture of ADSCs and CX_3_CR1^+^ lining macrophages at a 1:1 ratio. Scale bar, 400 μm.


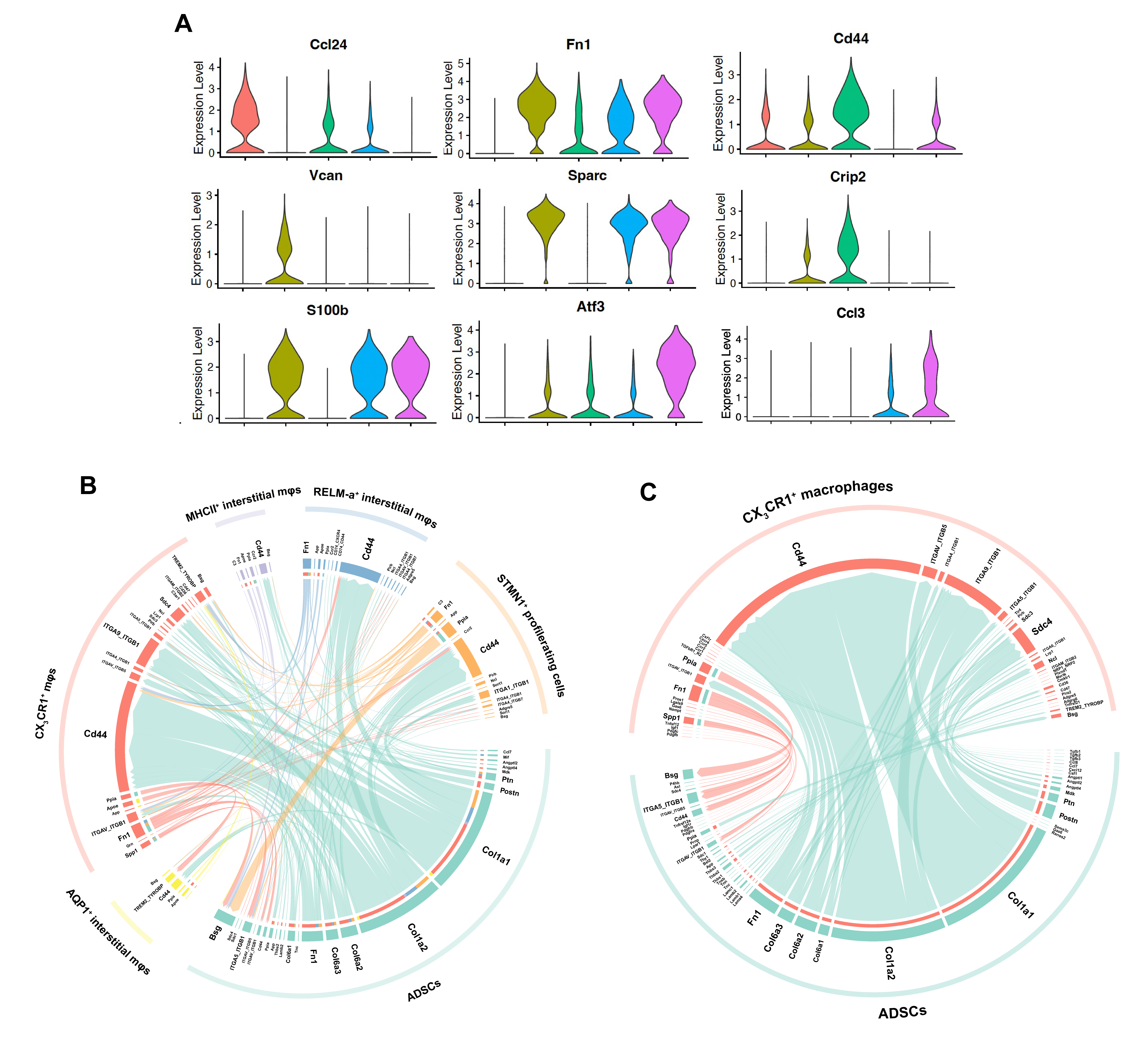


Fig. S4.

(A) Representative differential gene expression of the five CX_3_CR1^+^ lining macrophage subgroups. (B) CellChat chord diagram of cell-cell communications between ADSCs and synovial CD45^+^ CD11b^+^ LY6G^−^ cells based on the scRNA-seq results (Padj < 0.05, Prob > 1e-5). (C) CellChat chord diagram of cell-cell communication between ADSCs and CX_3_CR1^+^ lining macrophages based on the scRNA-seq results (Padj < 0.05, Prob > 1e-7).
